# PyBIDS: Python tools for BIDS datasets

**DOI:** 10.21105/joss.01294

**Published:** 2019-08-12

**Authors:** Tal Yarkoni, Christopher J Markiewicz, Alejandro de la Vega, Krzysztof J Gorgolewski, Taylor Salo, Yaroslav O Halchenko, Quinten McNamara, Krista DeStasio, Jean-Baptiste Poline, Dmitry Petrov, Valérie Hayot-Sasson, Dylan M Nielson, Johan Carlin, Gregory Kiar, Kirstie Whitaker, Elizabeth DuPre, Adina Wagner, Lee S Tirrell, Mainak Jas, Michael Hanke, Russell A Poldrack, Oscar Esteban, Stefan Appelhoff, Chris Holdgraf, Isla Staden, Bertrand Thirion, Dave F Kleinschmidt, John A Lee, Matteo Visconti di Oleggio Castello, Michael P Notter, Ross Blair

**Affiliations:** 1 University of Texas at Austin; 2 Stanford University; 3 Florida International University; 4 Dartmouth College; 5 University of Oregon; 6 McGill University; 7 University of Southern California; 8 Concordia University; 9 National Institute of Mental Health; 10 MRC Cognition and Brain Sciences Unit; 11 Montreal Neurological Institute and Hospital; 12 Alan Turing Institute; 13 Otto-von-Guericke University Magdeburg; 14 CorticoMetrics LLC; 15 Télécom ParisTech, France; 16 Max Planck Institute for Human Development, Berlin, Germany; 17 University of California at Berkeley; 18 Queen Mary University London; 19 INRIA; 20 Rutgers University; 21 University of Lausanne

## Summary

Brain imaging researchers regularly work with large, heterogeneous, high-dimensional datasets. Historically, researchers have dealt with this complexity idiosyncratically, with every lab or individual implementing their own preprocessing and analysis procedures. The resulting lack of field-wide standards has severely limited reproducibility and data sharing and reuse.

To address this problem, we and others recently introduced the Brain Imaging Data Standard (BIDS; (Gorgolewski et al., 2016)), a specification meant to standardize the process of representing brain imaging data. BIDS is deliberately designed with adoption in mind; it adheres to a user-focused philosophy that prioritizes common use cases and discourages complexity. By successfully encouraging a large and ever-growing subset of the community to adopt a common standard for naming and organizing files, BIDS has made it much easier for researchers to share, re-use, and process their data (Gorgolewski et al., 2017).

The ability to efficiently develop high-quality spec-compliant applications itself depends to a large extent on the availability of good tooling. Because many operations recur widely across diverse contexts—for example, almost every tool designed to work with BIDS datasets involves regular file-filtering operations—there is a strong incentive to develop utility libraries that provide common functionality via a standardized, simple API.

PyBIDS is a Python package that makes it easier to work with BIDS datasets. In principle, its scope includes virtually any functionality that is likely to be of general use when working with BIDS datasets (*i.e.*, that is not specific to one narrow context). At present, its core and most widely used module supports simple and flexible querying and manipulation of BIDS datasets. PyBIDS makes it easy for researchers and developers working in Python to search for BIDS files by keywords and/or metadata; to consolidate and retrieve file-associated metadata spread out across multiple levels of a BIDS hierarchy; to construct BIDS-valid path names for new files; and to validate projects against the BIDS specification, among other applications.

In addition to this core functionality, PyBIDS also contains an ever-growing set of modules that support additional capabilities meant to keep up with the evolution and expansion of the BIDS specification itself. Currently, PyBIDS includes tools for (1) reading and manipulating data contained in various BIDS-defined files (*e.g.*, physiological recordings, event files, or participant-level variables); (2) constructing design matrices and contrasts that support the new BIDS-StatsModel specification (for machine-readable representation of fMRI statistical models); and (3) automated generation of partial Methods sections for inclusion in publications.

PyBIDS can be easily installed on all platforms via pip (pip install pybids), though currently it is not officially supported on Windows. The package has few dependencies outside of standard Python numerical and image analysis libraries (*i.e.*, numpy, scipy, pandas, and NiBabel). The core API is deliberately kept minimalistic: nearly all interactions with PyBIDS functionality occur through a core BIDSLayout object initialized by passing in a path to a BIDS dataset. For most applications, no custom configuration should be required.

Although technically still in alpha release, PyBIDS is already being used both as a dependency in dozens of other open-source brain imaging packages – *e.g.*, fMRIPrep (Esteban et al., 2019), MRIQC (Esteban et al., 2017), datalad-neuroimaging (https://github.com/datalad/datalad-neuroimaging), and fitlins (https://github.com/poldracklab/fitlins) – and directly in many researchers’ custom Python workflows. Development is extremely active, with bug fixes and new features continually being added (https://github.com/bids-standard/pybids), and major releases occurring approximately every 6 months. As of this writing, 29 people have contributed code to PyBIDS, and many more have provided feedback and testing. The API is relatively stable, and documentation and testing standards follow established norms for open-source scientific software. We encourage members of the brain imaging community currently working in Python to try using PyBIDS, and welcome new contributions.
